# Three-dimensional bioprinting of mucoadhesive scaffolds for the treatment of oral mucosal lesions; an *in vitro* study

**DOI:** 10.1186/s41205-022-00157-5

**Published:** 2022-09-28

**Authors:** Maryam Koopaie, Duha Hayder Mohammad Ali Nassar, Mahvash Shokrolahi

**Affiliations:** 1 Department of Oral Medicine, School of Dentistry, Tehran University of Medical Sciences, Tehran, Iran; 2 Department of Oral Medicine, School of Dentistry, Tehran University of Medical Sciences, North Kargar St, P.O.BOX:14395 -433, Tehran, 14399-55991 Iran; 3 New Technologies Research Center, Amirkabir University of Technology (Tehran Polytechnic), Tehran, Iran

**Keywords:** 3D bioprinting, Drug delivery systems, Controlled release, Mucoadhesive scaffold, Scaffold

## Abstract

**Background:**

Chronic oral lesions could be a part of some diseases, including mucocutaneous diseases, immunobullous diseases, gastrointestinal diseases, and graft versus host diseases. Systemic steroids are an effective treatment, but they cause unfavorable and even severe systemic side effects. Discontinuation of systemic corticosteroids or other immunosuppressive drugs leads to relapse, confirming the importance of long-term corticosteroid use. The present study aims to fabricate a mucoadhesive scaffold using three-dimensional (3D) bioprinting for sustained drug delivery in oral mucosal lesions to address the clinical need for alternative treatment, especially for those who do not respond to routine therapy.

**Methods:**

3D bioprinting method was used for the fabrication of the scaffolds. Scaffolds were fabricated in three layers; adhesive/drug-containing, backing, and middle layers. For evaluation of the release profile of the drug, artificial saliva was used as the release medium. Mucoadhesive scaffolds were analyzed using a scanning electron microscope (SEM) and SEM surface reconstruction. The pH of mucoadhesive scaffolds and swelling efficacy were measured using a pH meter and Enslin dipositive, respectively. A microprocessor force gauge was used for the measurement of tensile strength. For the evaluation of the cytotoxicity, oral keratinocyte cells' survival rate was evaluated by the MTT method. Folding endurance tests were performed using a stable microsystem texture analyzer and analytic probe mini tensile grips.

**Results:**

All scaffolds had the same drug release trend; An initial rapid explosive release during the first 12 h, followed by a gradual release. The scaffolds showed sustained drug release and continued until the fourth day. The pH of the surface of the scaffolds was 5.3–6.3, and the rate of swelling after 5 h was 28 ± 3.2%. The tensile strength of the scaffolds containing the drug was 7.8 ± 0.12 MPa. The scaffolds were non-irritant to the mucosa, and the folding endurance of the scaffolds was over three hundred times.

**Conclusion:**

The scaffold fabricated using the 3D bioprinting method could be suitable for treating oral mucosal lesions.

## Background

Oral mucosal lesions are usually painful lesions associated with discomfort [1], leading to nutritional deficiencies in severe cases [2]. Oral ulcerative lesions can be divided into acute and chronic, depending on their manifestation and development [3]. Chronic oral lesions could manifest some diseases, including immunobullous diseases such as pemphigus vulgaris and graft-versus-host disease (GVHD) [4, 5]. Wound healing involves a sequence of complex biological processes performed in all tissues with the same pattern to complete the healing process with minimal scarring [6, 7]. The current treatment for oral ulcerative lesions involves using medications such as mouthwashes, creams, or ointments that are less effective because the medication does not have enough time to contact the lesion. In addition, common types of buccal drug delivery systems do not allow the patient to consume food and drink simultaneously, or in some cases, patients will complain of difficulty speaking [6, 8]. Topical corticosteroids can be used as adhesive vehicles or mouth rinses [9]. However, their therapeutic benefits are not always evident, and side effects are caused by topical corticosteroids, including secondary candidiasis, nausea, refractory response, mucosal atrophy, oral dryness, sore throat, unpleasant taste, and delayed healing [9, 10]. Some creams currently contain steroids, ointments, or pastes specially made, tested, and suitable for the oral cavity with limited efficacy [11, 12]. Besides this, the attachment and adhesion of most creams, ointments, and pastes to the saliva covering the oral mucosal surfaces is unfavorable mouth and tongue movements also increase their removal after application, resulting in short drug delivery times and being less effective [13]. Systemic steroids are more effective but cause severe systemic side effects [14]. Discontinuation of systemic corticosteroids leads to relapse, confirming the importance of long-term corticosteroid use [15]. These problems intensify the clinical need for alternative treatments, especially for those who do not respond to routine therapy; alternative treatments should have the ability to deliver controlled amounts of the drug locally to the lesion site [10, 16]. Oral mucosa is considered an important route for drug delivery [17]. Oral mucosa has arteries that are very permeable and accessible, allowing for systemic absorption of the drug painlessly and with a stable rate, bypassing the stomach and initial metabolism in the liver, which leads to an increase in its biological activity [13, 18]. The permeability of the oral mucosa is estimated to be about 4000 times that of the epidermis, which helps the drug be absorbed rapidly [19, 20]. Mucoadhesion is valid for drug delivery, such as tablets, patches, and gels [21]. Benefits of mucoadhesive scaffolds include increased residence time at application sites, drug protection, increased drug penetration, and enhanced drug availability [22, 23]. Using a mucoadhesive membrane will reduce toxicity, maximize the drug dose to the lesion, and minimize dose [24]. Mucoadhesive improves the advantage of drug localization in the affected region [25]. There are various systems for drug delivery to the mucosal layer, including adhesive tablets, gels, and, more recently, films, which have been developed. Buccal films are superior to sticky tablets in terms of flexibility and comfort. In addition, buccal films are suitable for protecting wound surfaces, reducing pain, and increasing the effectiveness of treatment [26, 27]. Although Nesic et al. reviewed three-dimensional (3D) printing techniques for tissue regeneration and oral vascular rehabilitation [28], limited studies have been performed on oral drug delivery to treat oral lesions through 3D printing [29]. Regarding limited data and studies about treating oral mucosal lesions with mucoadhesive scaffolds and the lack of standardization of these techniques, this study aims to fabricate a mucoadhesive scaffold by 3D bioprinting for drug delivery systems to treat oral mucosal lesions.

## Methods

### Ethical statement

This study was approved by the Tehran University of Medical Sciences Ethical Committee (ethical code: IR.TUMS.DENTISTRY.REC.1399.100). All methods were performed in accordance with the relevant guidelines and regulations.

### Preparation of bio-ink

#### Single layered scaffold

The scaffolds in the first step were fabricated in one layer, and for the fabrication of the scaffolds and preparation of the biological ink, alginate was selected as the base material for preparing patches containing the drug. Regarding the alginate properties in mucosal adhesion and the ability to slow drug release [30, 31], alginate was selected as the scaffold polymer base, and calcium sulfate was used to cross-link alginate partially and improve its printability [32]. For this purpose, according to Table 1, solutions were prepared, and their printability was checked. Since the compounds proposed using CaSO_4_ were not uniform enough and did not show good printability, the 3.0% gelatin and CaCl_2_ as cross-linkers will improve the printability.

**Table 1:** Chemical composition of drug-containing inks

**Solvent**	**Concentration**		**Crosslinker**	**BioInk: Crosslinker**
	**Alginate**	**Drug**		
Deionized water	8.0%	0.8%	2.5% CaSO_4_	10:1
				8:1
Deionized water	6.0%	0.8%	2.5% CaSO_4_	10:1
				8:1
Deionized water	7.0%	0.8%	5.0% CaCl_2_	10:1
				8:1

#### Three-layered scaffold

After fabricating a single-layered scaffold, we fabricated the scaffolds in three layers because of the need for a hydrophobic layer to protect the scaffold from the oral cavity. The three-layered scaffold is composed of the bottom layer, which is a hydrophobic layer of ethylcellulose that protects the scaffold in the oral cavity. The middle layer combines ethylcellulose and hydroxypropyl methylcellulose (HPMC) that holds the bottom layer and the top layer to be printed together. The top layer containing the drug is alginate, gelatin, and HPMC (Table 2).

**Table 2:** Chemical composition of improved solutions as drug-containing inks

	**Material**	**Concentration**	**Crosslinker** **(Type and concentration)**	**Solvent**
**Bottom layer**	Ethylcellulose		5.0% CaCl_2_	Deionized water
**Middle layer**	Ethylcellulose & HPMC			
**Top layer**	Alginate	7.0%		
	Gelatin	3.0%		
	HPMC	0.8%		
	Drug	0.8%		

#### Three dimensional (3D) bioprinting of oral scaffolds

**Fig. 1:**
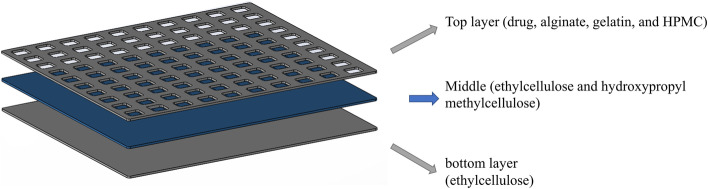
Schematic of three-layered scaffold design

The 3D bioprinting method was used for the fabrication of the scaffolds. A mesh-like design was used for 3D bioprinting (20 × 20 × 2 mm dimensions) to print the oral scaffold. Printing geometry and characteristics of the single-layered and three-layered scaffolds (Fig. 1) are shown in Tables 3 and 4, respectively.

**Table 3:** Printing geometry and characteristic of the single-layered scaffold

**Print geometry**	**The diameter of the nozzle**	0.5 mm
	**The distance between two strings**	2.2 mm
	**Dimensions**	20 × 20 × 2 mm
	**Print design**	
**Print conditions**	**Temperature**	25 ^O^C
	**Pressure**	0.3 bar
	**Print speed**	15 mm/s
	**Layer height**	Each layer is 100 µm

**Table 4:** Printing geometry and characteristics of the three-layered scaffold

**Print geometry**	**The diameter of the nozzle**	0.5 mm
	**The distance between two strings**	2.2 mm
	**Dimensions**	20 × 20 × 2 mm
	**Print design of top layer**	
**Print conditions**	**Temperature**	25 ^O^C
	**Pressure**	0.3 bar
	**Print speed**	15 mm/s
	**Layer height**	Each layer is 50 µm

### Evaluation of *in vitro* drug profile release

#### Betamethasone

Artificial saliva was selected as the release medium to evaluate the drug release profile. For this purpose, first, specific concentrations of betamethasone as a drug were prepared in artificial saliva, and after reading their absorption rates at 190 to 500 nm NanoDrop (BOECO Micro UV–VIS, Germany), a graph of the absorption rate was drawn according to the concentration. The printed scaffold was placed in 20 ml of artificial saliva in a 37 °C incubator shaker to simulate physiological conditions. At regular intervals, 1 ml of the release medium was removed, and the same amount of fresh saliva was added to the release medium. After absorption, samples were taken from the release medium and were read at a wavelength of 246 nm and calculated using the standard diagram and Eq. 1 [33]. The cumulative value of the drug released from the sample was calculated.

1 $$\mathrm{Er}(\%)=\;\left(\frac{\mathrm{Vo}\times\mathrm{Cn}+\mathrm{Vr}\times{\mathrm\Sigma1}^{{}^{n-1}{}}\mathrm{Ci})}{\mathrm{M\;total}}\right)\times100$$

M_total_: The total amount of drug trapped in the sample

V_o_: Volume of the release medium

V_r_: Volume of the alternative medium

C_n_: Drug concentration in the samples

#### Prednisolone

In the first step, before examining the ability to release prednisolone from multilayer scaffolds externally, its standard concentration diagram was drawn. For this purpose, artificial saliva solutions were considered as a receptor phase (release medium) in drug release studies. Artificial saliva solutions containing different amounts of the drug were prepared and after normalizing the device compared to the artificial saliva solution, the drug absorption at the maximum wavelength (243 nm) was determined by NanoDrop (BOECO Micro UV–VIS, Germany). This experiment was repeated for three days, and the standard graph of mean absorption was plotted as a function of their concentration in the saliva solution.

In order to evaluate the amount of drug released from the prepared scaffolds, specific dimensions (15 mm × 15 mm) of each scaffold with a specific weight were immersed in jars containing 4 ml of artificial saliva solution and then incubated at 37 °C; This operation was repeated three times for each scaffold. At specified times, the solution inside the jars was transferred to the NanoDrop machine tank and immediately replaced with a fresh saliva solution. After measuring the amount of absorption by the NanoDrop device, the amount of absorption was converted to concentration using a standard concentration chart, and the average amount of the drug was calculated by repeating the measurement of its release. Then, the cumulative release percentage of the drug (Er%) was calculated by Eq. 1, and the cumulative release diagram of the drug was plotted as a function of incubation time. The scaffolds were printed using BioFab X2 machine (3D BIO, Omidafarinan company, Iran) at 25◦C during the printing. Printed scaffolds were cured using the UltraViolet-C (UV-C) lamp for 4 min.

### Scanning electron microscopy (SEM) and 3D surface reconstruction

SEM images were taken using scanning electron microscopy (ZEISS EVO MA 25, Zeiss Evo 25, Germany). Images were prepared with a voltage of 5–10 kV. Samples were dehydrated before imaging. The specimens were connected to a 5 mm diameter stub pin using a 350 mm diameter pin. The samples were coated with gold using a sputtering method for 2 min at 15 Ma (JEOL Ltd., Tokyo, Japan) under an argon atmosphere, and images were taken at different resolutions. To evaluate the surface topography of scaffolds, 3D surface reconstruction (3DSM, Carl Zeiss scanning electron microscopy) and MountainsSEM® were used.

### PH evaluation

The pH of mucoadhesive scaffolds was measured using a pH meter, and the sample with dimensions of 10 cm^2^ was dissolved in 10 ml of water. This process was repeated three times for each sample.

### Folding endurance test

A folding endurance test assesses the scaffold's ability by folding and bonding the scaffold without breaking or cracking it. Folding endurance tests were performed using a stable microsystem texture analyzer and analytic probe mini tensile grips. A folding endurance test was performed by bending the scaffold several times, up to 300 times, until the scaffold was torn.

### Swelling efficacy profile measurement

It was measured by using Enslin dipositive. The samples were exposed to a buffer solution at pH 6.8 for 5 h. The dimensions of the scaffold used were 1 cm^2^. Our scaffold was placed on a sintering filter, and the volume of material absorbed by the samples was measured by pipetting after 5 h. The volume of fluid absorbed by the samples was recorded.

### Evaluation of cytotoxicity

To evaluate the toxicity of the scaffold, oral keratinocyte cells cultured from DMEM medium were cultured in 6-well plates (3 × 10^5^ cells per well). The cells were incubated for 24 h; Then the cells were examined in 3 groups: 1- Cells in the vicinity of the drug-free scaffold, 2- Cells in the vicinity of the drug-containing scaffold, and 3- Cells in the control group. 4.5 ml of culture medium was added to each group. After 24 h of cell incubation in the vicinity of the film, the rate of cell survival in 3 groups was evaluated by 4,5-Dimethylthiazol-2-yl) -2,5-Diphenyltetrazolium Bromide (MTT) method. Each group was repeated three times, and the mean value (95% CI) was reported.

### Measurement of tensile strength

A microprocessor force gauge and a scaffold evaluated the tensile strength using SANTAM-STM50 (SANTAM-Eng. Design Co. LTD) with the force–displacement (F–D) and stress–strain (σ–ε) diagram (ASTM D1708). The scaffold was mounted between the upper and lower clamps. The upper clamp was moved at 2 mm/min speed until the scaffold was torn. The drug-free and drug-containing scaffold tensile strength was recorded based on the number displayed in the force gauge.

## Results

### Printability

#### Single layered scaffold

**Fig. 2:**
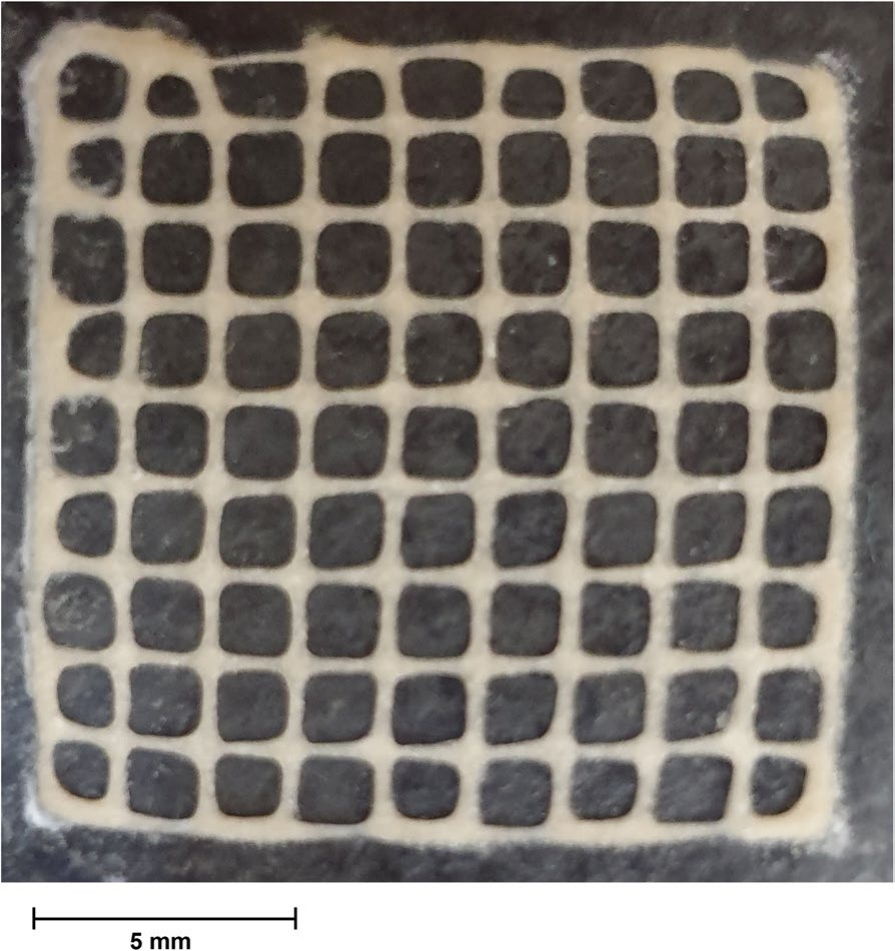
Macro image of a 3D printed scaffold

By examining different formulations, it was observed that the combination of 7wt% alginate and 3wt% gelatin was found to have good printability after evaluating various formulas. As the number of layers increased and the ink warmed up, the material dispersed, and the layers collapsed. Macro image of a 3D printed scaffold is shown in Fig. 2.

#### Three-layered scaffold

**Fig. 3:**
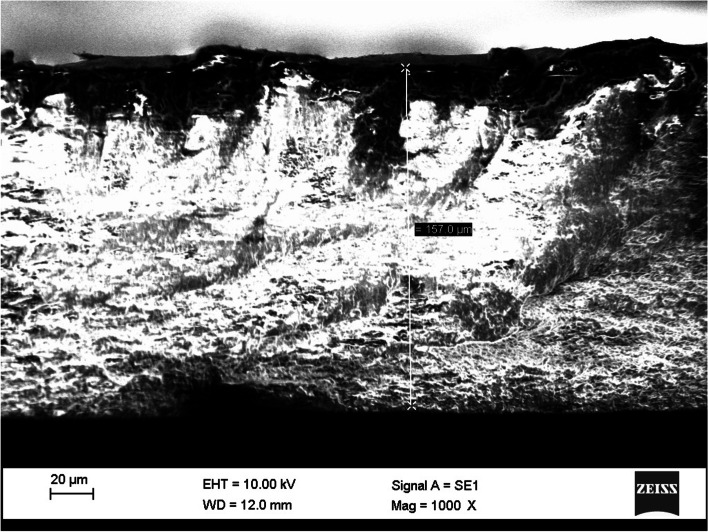
Cross-section view of three-layered scaffold (Magnification = 1000 X)

**Fig. 4:**
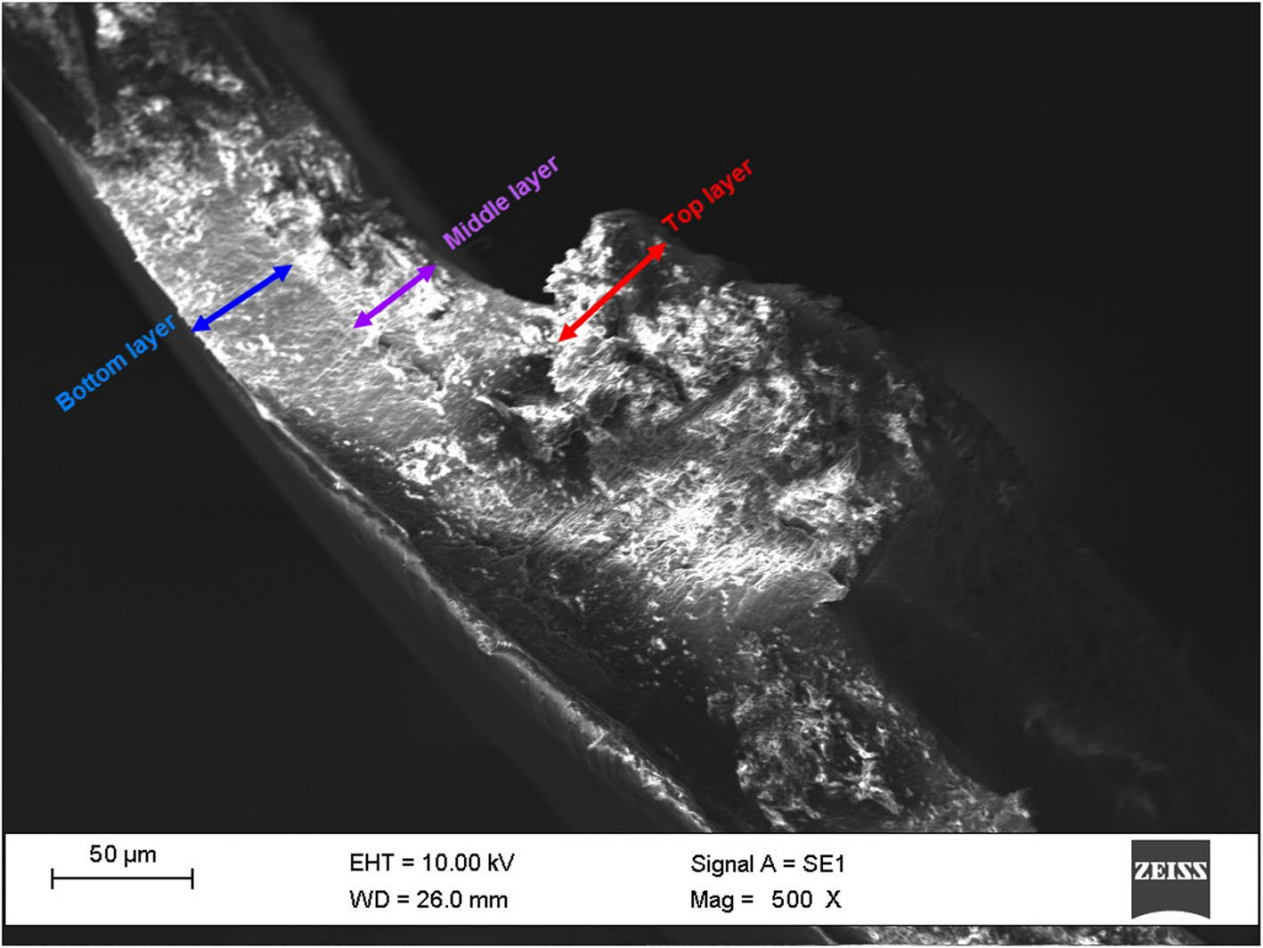
Cross-section view of three layered scaffold (Magnification = 500 X)

By examining the different formulations that were described in the material section, it was observed that the combination of 7 wt% alginate, 3 wt% gelatin, 0.8 wt% HPMC, and drug (based on the weight of polymers) for the top layer has acceptable printability. Cross-section view of scaffolds with different magnifications are illustrated in Figs. 3 and 4.

As shown in Fig. 3, the thickness of the three layers is about 150 µm, and Fig. 4 depicts that the thickness of each layer is 50 µm, confirming the desired thickness of scaffolds.

**Fig. 5:**
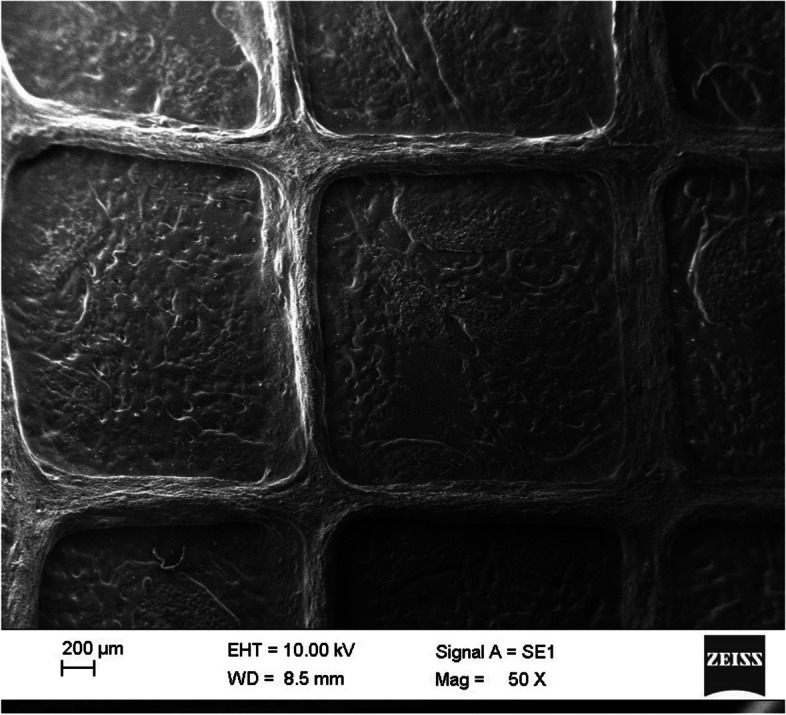
SEM image of 3 layered scaffold (Magnification = 50 X)

**Fig. 6:**
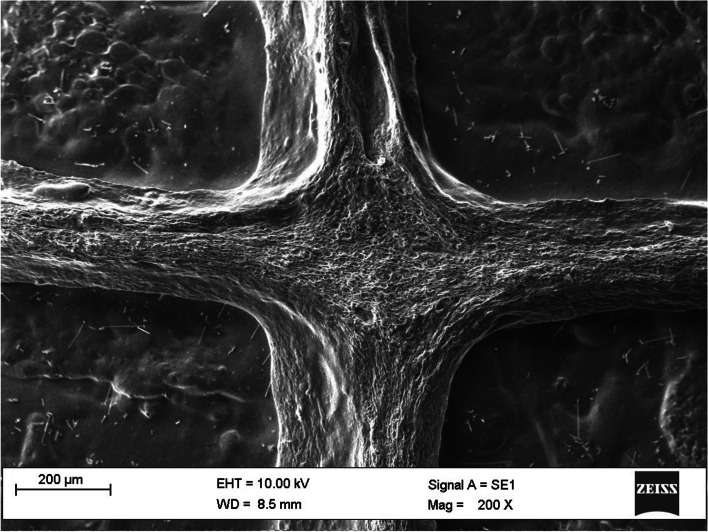
SEM image of 3-layered scaffold (Magnification = 200 X)

SEM images of 3-layered scaffold surface are shown in Fig. 5 and Fig. 6.

**Fig. 7:**
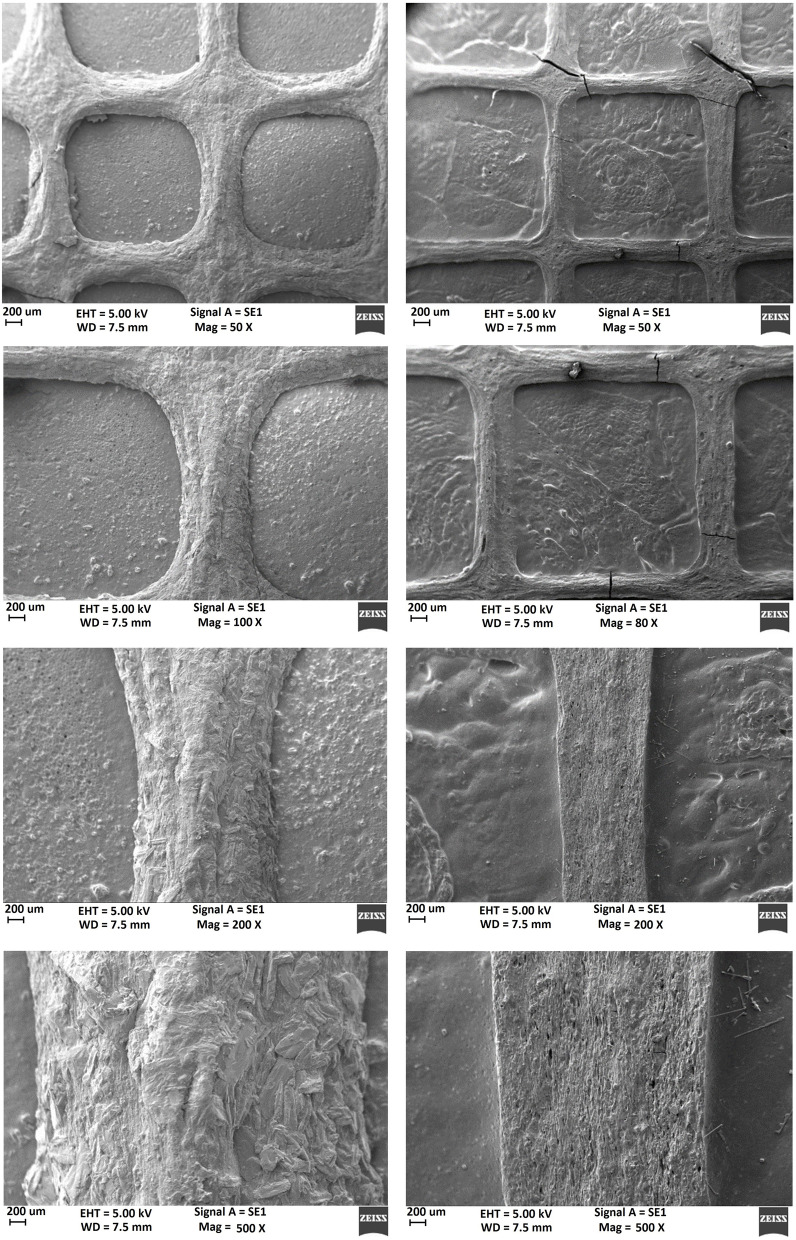
SEM image of 3-layered scaffold surface (Magnification = 50 to 500 X)

SEM images of 3-layered scaffold surface with a magnification of 50 to 500 are shown in Fig. 7.

**Fig. 8:**
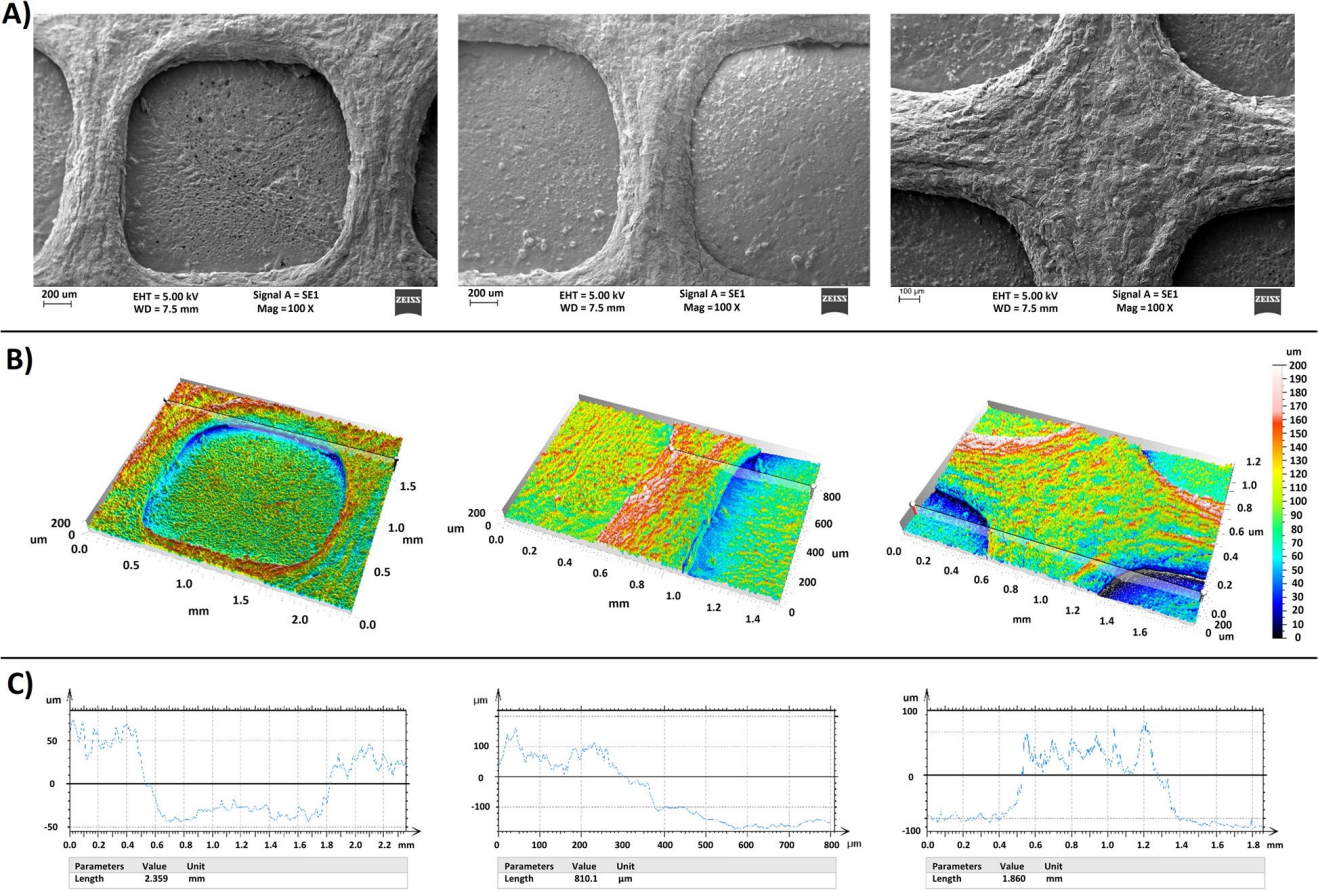
**A)** SEM images, **B) **surface topography, and **C)** line scan of surface roughness of 3d-printed scaffolds

SEM images (Fig. 8-A), surface topography (Fig. 8-B), and line scan of surface roughness (Fig. 8-C) of scaffolds using 3D surface reconstruction (3DSM, Carl Zeiss scanning electron microscopy) and MountainsSEM® are shown in Fig. 8.

### pH and folding endurance measurement results

The pH of the scaffold surface was in the range of 5.3–6.3. This range is close to the normal pH of the oral cavity, and therefore it does not irritate the mucosa. The folding endurance of the scaffold is more than 300 times.

### Swelling index and Tensile strength

The swelling index of the oral scaffold should ensure the patient's comfort. Excessive swelling leads to discomfort to the patients and results in removing the scaffold from the mucosa. Swelling further causes faster and uncontrollable drug release. Swelling further causes faster and uncontrollable drug release. The rate of swelling after 5 h was 28 ± 3.2%. The tensile strength of the scaffold without the drug was 7.1 ± 0.34 MPa and 7.8 ± 0.12 MPa in the scaffold containing the drug.

### Evaluation of Cytotoxicity

**Fig. 9:**
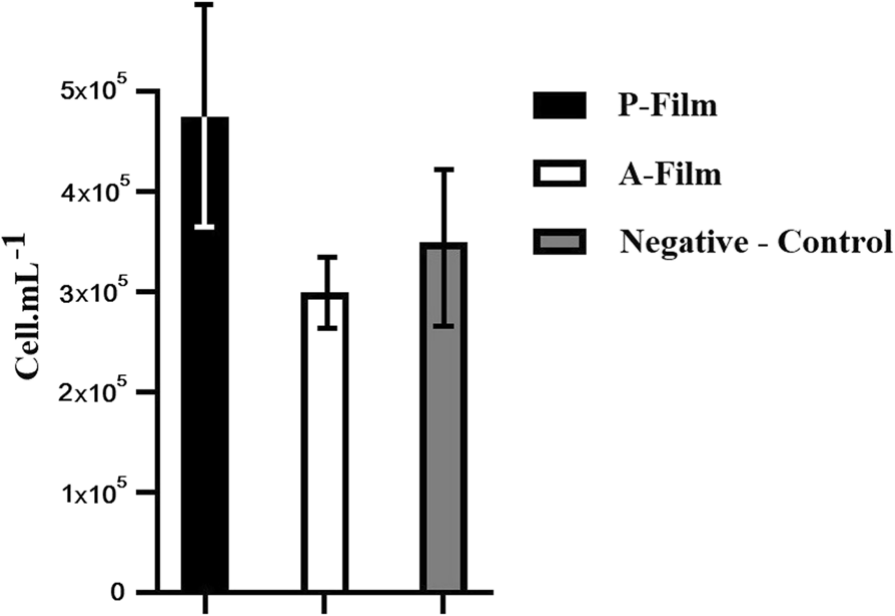
Chart of cytotoxicity evaluation of scaffolds. P-Film is a drug-free scaffold, while A-Film is a drug-containing scaffold

MTT results showed that after 1 h of incubation, drug-free scaffolds did not reduce cell viability compared to the control group, and therefore it can be considered non-irritant (Fig. 9).

### Drug Release

#### Betamethasone

**Fig. 10:**
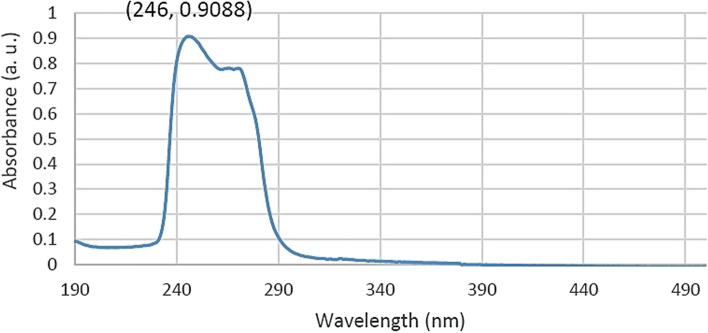
Absorption spectrum of different concentrations of betamethasone in artificial saliva

An absorption spectrum was first taken from the betamethasone drug between 190 and 800 nm to prepare the standard diagram and release profile (Fig. 10).

**Fig. 11:**
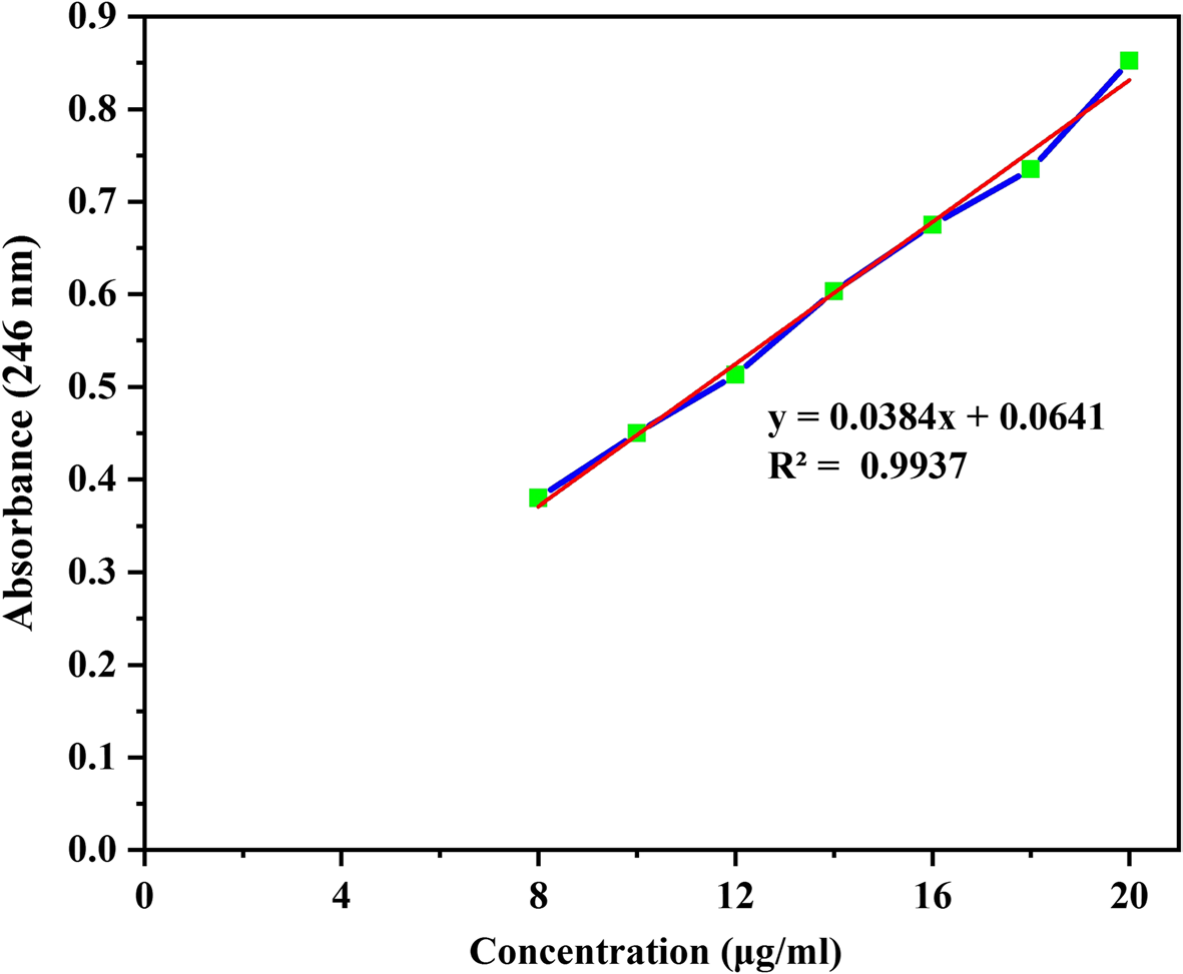
Standard chart of betamethasone in artificial saliva

In the absorption spectrum of betamethasone, it was observed that there was a peak at the wavelength of 246 nm. Therefore, this wavelength was chosen as an identifier to draw the standard diagram and read the absorption of release samples. After the scaffold containing the drug was printed and placed in the release medium and the incubator, the sample was taken at different hours. Finally, the absorbance of the samples at 246 nm was read. The concentration of drugs released into the release medium was calculated by placing the absorption numbers in the formula obtained in the standard diagram. To calculate the cumulative percentage of the drug, the weight of the printed scaffold was first measured. By establishing the ratio between the weight of the scaffold and the weight of one milliliter of unprinted ink, the volume of ink printed with a drug concentration of 0.8%, the resulting amount of drug in the scaffold was obtained (Fig. 11).

According to calculations, 148.304 micrograms of the drug were placed in the release environment in the printed scaffold. The following chart was drawn by placing the concentrations obtained from the standard chart in the cumulative percentage formula (Table 5).

**Table 5:** Concentration and cumulative percentage of drug released into the release medium at different hours

**Time (hour)**	**∑C (ug/ml)**	**Cumulative release (%)**
**1**	0.017	2.304
**2**	0.018	2.674
**3**	0.019	2.930
**4**	0.019	3.058
**5**	0.021	3.471
**24**	0.057	8.749
**72**	0.059	9.415
**144**	0.064	10.450
**216**	0.136	21.087

**Fig. 12:**
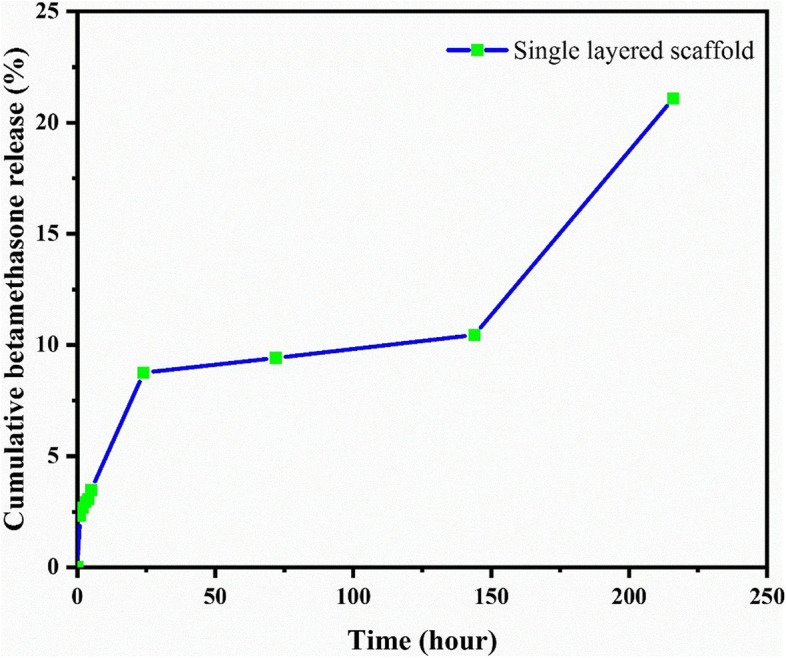
Diagram of the cumulative release of betamethasone in artificial saliva

As seen in the cumulative release chart, after nine days, about 21% of the drug in the printed scaffold was released into the environment (Fig. 12).

### Repeated release test

**Fig. 13:**
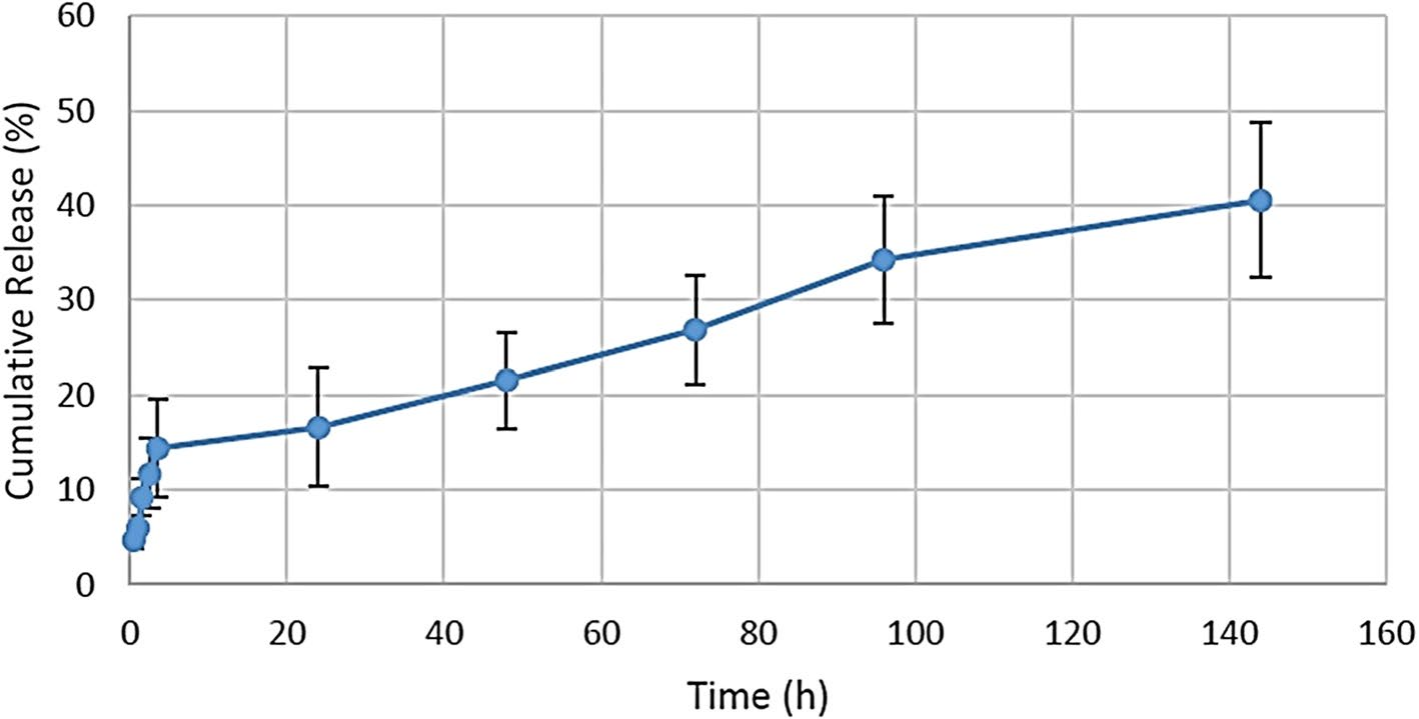
Graph of cumulative percentage of drug released into the release medium during one week

The ink containing the drug was prepared by combining 5wt% gelatin, 7wt% by weight alginate, and 1.2 mg/ml betamethasone to repeat the test. With drug-free ink and drug-containing ink, all three scaffolds were printed with the design mentioned in the previous test and placed in 5wt% calcium chloride for 15 min to cross-link. The scaffolds were then placed in a Falcon containing 20 ml of artificial saliva and transferred to a sugar incubator at 37 °C. After the scaffolds were placed in the release medium, and inside the incubator, samples were taken at different hours, and finally, the absorption of the samples was read at 246 nm. At each hour, a sample is taken from the environment where the scaffold was without medication; the device was first reset to zero. The concentration of drug released into the release medium was calculated by placing the adsorption numbers into the formula obtained in the standard diagram. To calculate the cumulative percentage of the drug, the weight of the printed scaffold was first measured by establishing the ratio between the weight of the scaffold and the weight of one milliliter of unprinted ink, the volume of ink that was printed. Then the amount of drug present in the scaffold was obtained. According to calculations, there was an average of 0.115 mg of drug per scaffold. By placing the concentrations obtained from the standard graph in the cumulative percentage Eq. 2, and cumulative drug release was calculated (Fig. 13).

2 $$\mathrm{Er}(\%)\;=\;\left(\frac{\mathrm{Vo}\times\mathrm{Cn}+\mathrm{Vr}\times{\mathrm\Sigma1}^{n-1}\mathrm{Ci}}{\mathrm{Mtotal}}\right)\times\;100\;$$

After nine days, when we used Betamethasone as a drug, only about 21% of the drug in the printed scaffold was released into the environment. We wanted to reach an optimum concentration in the release test we took, and Betamethasone had a low concentration; therefore, we changed the drug to Prednisolone 50 mg.

#### Prednisolone

**Fig. 14:**
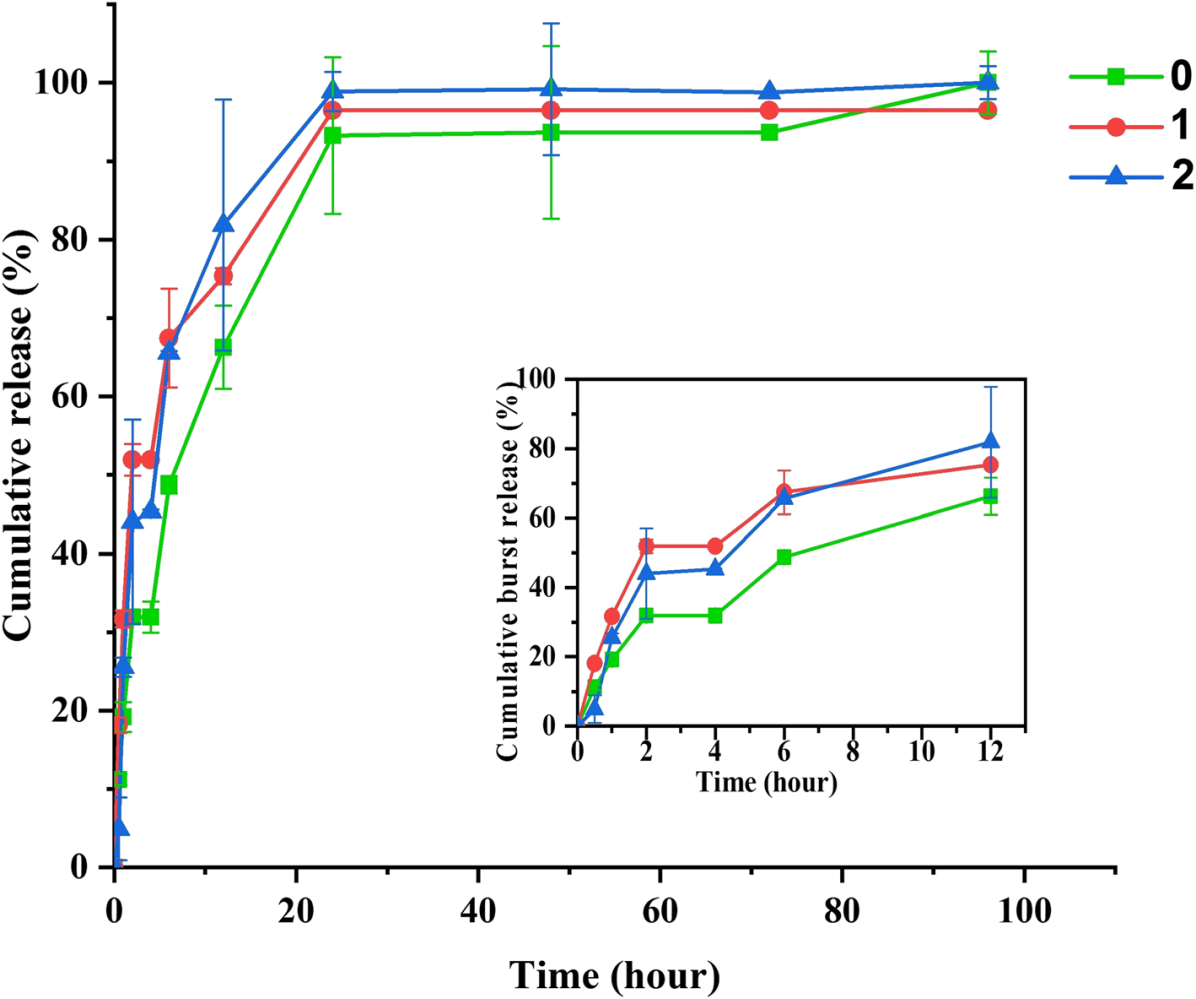
Cumulative release profiles of three-dimensional scaffolds over time, with explosive release charts in the early hours

Figure 14 shows the cumulative release and the explosive release of the three-layered scaffolds. All scaffolds had the same drug release curve: an initial rapid explosive release during the first 12 h, followed by a gradual release. The three scaffolds (0, 1, and 2) were released respectively 66.27%, 75.34%, and 81.88% of their loaded drugs during the first 12 h. Scaffold 2 had a smaller diameter than the other two, and as the diameter decreases due to the increase in the sample's surface area, more drug molecules are in contact with the release medium, causing a higher drug to be released during the first hours. After 24 h, the entire loaded drug was released. Scaffold 0 had a slower release rate, and the initial slope of its release diagram was lower than the other two scaffolds, and the release of the drug continued until the fourth day (Fig. 14).

Scaffold containing Prednisolone could release all the drugs, and even the scaffold was disintegrated. We could reach to high drug concentration release that we could not reach with Betamethasone.

## Discussion

The present study aimed to design a mucosal scaffold by 3D bioprinting and gradual release of the drug into the oral environment. The 3D bioprinting method is an accurate and practical method for making bio-scaffolds. In the present study, a mucoadhesive scaffold consisting of three layers was fabricated, which contained a bioadhesive layer and a hydrophobic protective backing layer. These scaffolds showed acceptable mechanical and biological properties. The pH of the fabricated scaffolds was 5.3—6.3, similar to the saliva pH (5.6—7.9) [34, 35]. This pH does not cause irritation or tissue damage. Successful bio-adhesion depends on the rapid hydration of scaffolds [36]. The scaffolds in the present study show rapid swelling, which is an acceptable feature for scaffolds. Another feature of the fabricated scaffold in the present study was sustained drug release, while most similar studies did not have this feature [37–40].

Santocildes-Romero et al. fabricated mucoadhesive membranes by electrospinning to treat mucosal lesions such as oral lichen planus lesions [41]. They used a double-layered (adhesive/drug-containing layer and a backing layer) membrane, while ours, a three-layered scaffold (protective layer/middle layer/drug-containing layer) was fabricated. They attached the layers using the melting method, while we used CaCl_2_ cross-linker to attach the layers that other studies showed that CaCl_2_ cross-linked could be used for prolonging drug release [42]. The pH of membranes in the study by Santocildes-Romero et al. was 8.2, which is slightly more alkali than normal saliva [35], but the pH of the scaffold surface of our study was in the desired range: (5.3–6.3) and close to the normal pH. Therefore, it does not irritate the mucosa and any changes in saliva could lead to oral microbiome imbalance and diseases [43, 44]. All their patches increased in weight to approximately 70% of their weight within 60 min, but the swelling rate in our study was only 28 ± 3.2% after 5 h. Excessive swelling leads to discomfort in the patient and results in the patch's removal from the mucosa. Swelling further causes faster and uncontrollable drug release [45]. Their results showed rapid drug release [41] while ours showed sustained drug release, which is advantageous to have increased drug interpenetration and longer time of drug contact to the lesions. In our study, the release of the drug continued until day four, but in this study, the drug release continued only for 6 h, which is a fast drug delivery time [33, 36].

Tonglairoum et al. produced a one-layer scaffold of clotrimazole-microemulsion-containing nanofibers to treat oral candidiasis by electrospinning method [38]. Although their scaffolds showed low toxicity, *in vitro* drug release showed rapid drug release and lasted for only 24 h, but our *in vitro* results showed sustained drug release, and the release continued until the fourth day, which shows longer drug delivery to the lesions.

Tonglairoum et al. used a hybrid polyvinylpyrrolidone/hydroxypropyl β-cyclodextrin (PVP / HPβCD) nanofiber mat composed of clotrimazole (CZ) fabricated a one-layer with low toxicity to treat oral candidiasis [39]. This study showed rapid drug dissolution and release, while our results showed a gradual and sustained drug release, resulting in a longer drug delivery time. In another study, Tonglairoum et al. produced sandwich nanofibers composed of CZ using electrospinning. This drug showed rapid drug release, which differs from our goal, which was sustained drug release [40].

Alves et al. make patches to treat oral mucosal infections, especially targeted drugs for mucositis. They fabricated a bi-layered patch containing lidocaine, and the results showed rapid drug release. The mucoadhesive films fabricated in this study were biologically safe [37]. Hajikhani et al. manufactured composite nanofiber encapsulated collagen and cefazolin dressing scaffold using a coaxial electrospinning method to release the encapsulated composite [46]. The release rate of Cefazolin from their scaffold was such that it could be used for several days. Their scaffold had no non-polar backing layer to create a unilateral drug release to prevent Cefazolin's migration to the other side of the scaffold, but our scaffolds contained a backing layer for unilateral drug release and protection from the oral cavity. A fibrous membrane using electrospinning from an ethanol/water mixture was fabricated by Edmansa et al. The resulting fibrous membrane released the protein at a clinically desirable rate, reaching a cumulative release of 90 ± 13% after 2 h. An additional protective poly (caprolactone) backing layer was introduced to facilitate unidirectional transfer without loss of enzymatic activity [47].

Davoudi et al., a chitosan/gelatin/keratin composite containing sodium hydrocortisone succinate was formed by casting as a buccal mucosal adhesive patch to treat desquamative gingival inflammation was developed. Ethylcellulose was used as a backing layer to control the release rate. *In vitro* drug release showed a burst effect occurring in the first 3–4 h, which releases about 80% of the drug concentration. Based on the interactions between keratin and drug particles, some drugs were trapped in the system even after 120 h [48]. Alopaeus et al. prepared a double-layered buccal film using the solvent casting method. This study showed rapid drug release, different from our study results [49]. Szabo et al. manufactured nanofiber buccal film for antifungal agents *in vitro* and silico study [50]. Buccal films were fabricated by the electrospinning technique. The results show that the dissolution process takes place quickly and thoroughly, resulting in rapid drug delivery, which differs from the results of our study, which showed sustained drug release.

The scaffolds used in this research demonstrate fast swelling and sustained drug release, both of which were difficult to achieve in previous investigations [37–40]. Oral mucosal diseases such as lichen planus, pemphigus, and pemphigoid are chronic diseases requiring long-term corticosteroid treatment [51]. Long-term use of high doses of topical corticosteroids can lead to adrenal and hypothalamic–pituitary–adrenal axis suppression [52, 53].

Several limitations to this study need to be acknowledged. Although it has been tried to simulate the physiological conditions of the oral cavity, the scaffolds are immersed in different solutions, so our test conditions do not accurately reflect the physiological conditions of the oral cavity. A significant challenge and limitation were to study the combination of biological inks and drugs so that the properties of printing and drug release can be provided together.

In comparison, the gradual and continuous release of the drug from the patch reduces the final cumulative dose of the drug and reduces the likelihood of adrenal suppression. The lesions could be associated with a pain sensation; applying mucoadhesives may protect the lesions from external stimuli.

## Conclusion

This study designed and fabricated a three-layer mucoadhesive scaffold comprising a hydrophilic layer. The scaffolds showed sustained drug release into the environment. A hydrophobic protective backing layer protects the mucosa, and a middle bioadhesive layer is attached to the two layers. Our results showed that the 3D bioprinting method could be used to fabricate an oral mucoadhesive scaffold with acceptable mechanical properties for oral drug delivery of oral diseases. The presence of enzymes, temperature changes, and exposure to foods affect the rate of degradation and release of the drug into the oral cavity. For this reason, it is necessary to evaluate the scaffolds containing the drug *in vivo* as the next step.

## Data Availability

Any data or results regarding this study are available from the corresponding author on reasonable request.

## Abbreviations

3D: Three-dimensional
CZ: Clotrimazole
GVHD: Graft-versus-host disease
HPMC: Hydroxypropyl methylcellulose
MTT: 4,5-Dimethylthiazol-2-yl) -2,5-Diphenyltetrazolium Bromide
PVP/HPβCD: Hybrid polyvinylpyrrolidone/hydroxypropyl β-cyclodextrin
SEM: Scanning electron microscope
UV-C: UltraViolet-C
